# Development of compact DOI-measurable PET detectors for simultaneous PET/MR Imaging

**DOI:** 10.1186/2197-7364-2-S1-A2

**Published:** 2015-05-18

**Authors:** Yiping Shao, Xishan Sun, Kai Lou

**Affiliations:** University of Texas MD Anderson Cancer Center, USA; Rice University, USA

It is critically needed yet challenging to develop compact PET detectors with high sensitivity and uniform, high imaging resolution for improving the performance of simultaneous PET/MR imaging, particularly for an integrated/inserted small-bore system. Using the latest “edge-less” SiPM arrays for DOI measurement using the design of dual-ended-scintillator readout, we developed several compact PET detectors suited for PET/MR imaging. Each detector consists of one LYSO array with each end coupled to a SiPM array. Multiple detectors can be seamlessly tiled together along all sides to form a large detector panel. Detectors with 1.5x1.5 and 2.0x2.0 mm crystals at 20 or 30 mm lengths were studied. Readout of individual SiPM or capacitor-based signal multiplexing was used to transfer 3D interaction position-coded analog signals through flexible-print-circuit cables to dedicated ASIC frontend electronics to output digital timing pulses that encode interaction information. These digital pulses can be transferred to, through standard LVDS cables, and decoded by a FPGA-based data acquisition positioned outside the MRI scanner for coincidence event selection. Initial detector performance measurement shows excellent crystal identification even with 30 mm long crystals, ~18% and 2.8 ns energy and timing resolutions, and around 2-3 mm DOI resolution. A large size detector panel can be scaled up with these modular detectors and different PET systems can be flexibly configured with the scalable readout electronics and data acquisition, providing an important design advantage for different system and application requirements. It is expected that standard shielding of detectors, electronics and signal transfer lines can be applied for simultaneous PET/MR imaging applications, with desired DOI measurement capability to enhance the PET performance and image quality.

